# Neural Field Models with Threshold Noise

**DOI:** 10.1186/s13408-016-0035-z

**Published:** 2016-03-02

**Authors:** Rüdiger Thul, Stephen Coombes, Carlo R. Laing

**Affiliations:** Centre for Mathematical Medicine and Biology, School of Mathematical Sciences, University of Nottingham, University Park, Nottingham, NG7 2RD UK; Institute of Natural and Mathematical Sciences, Massey University (Albany), Private Bag 102-904, North Shore Mail Centre, Auckland, New Zealand

**Keywords:** Stochastic neural field, Interface dynamics, Fronts, Bumps, Non-Gaussian quenched disorder

## Abstract

The original neural field model of Wilson and Cowan is often interpreted as the averaged behaviour of a network of switch like neural elements with a distribution of switch thresholds, giving rise to the classic sigmoidal population firing-rate function so prevalent in large scale neuronal modelling. In this paper we explore the effects of such *threshold noise* without recourse to averaging and show that spatial correlations can have a strong effect on the behaviour of waves and patterns in continuum models. Moreover, for a prescribed spatial covariance function we explore the differences in behaviour that can emerge when the underlying stationary distribution is changed from Gaussian to non-Gaussian. For travelling front solutions, in a system with exponentially decaying spatial interactions, we make use of an interface approach to calculate the instantaneous wave speed analytically as a series expansion in the noise strength. From this we find that, for weak noise, the spatially averaged speed depends only on the choice of covariance function and not on the shape of the stationary distribution. For a system with a Mexican-hat spatial connectivity we further find that noise can induce localised bump solutions, and using an interface stability argument show that there can be multiple stable solution branches.

## Introduction

The study of waves, bumps and patterns in models of Wilson–Cowan type [1] is now a very mature branch of mathematical neuroscience, as discussed in the review by Bressloff [2], with many practical applications to topics including working memory, visual processing, and attention. For a recent and comprehensive description of neural fields and their applications we refer the reader to the book [3]. It is only relatively recently that stochastic effects in neural fields have begun to be considered, with important applications to problems such as binocular rivalry waves [4] and perceptual switching [5]. These stochastic models are often obtained by considering the addition of noisy currents (notionally a “Gaussian random noise”) to standard (deterministic) neural fields, and the resulting models are cast as stochastic nonlinear integro-differential equations driven by a Wiener process, such as in [6–14]. A rigorous probabilistic framework in which to study these equations has recently been provided by Faugeras and Inglis [15]. The analysis of patterns, waves and bumps in such models has been possible utilising tools from stochastic centre manifold theory (especially tools for weak noise analysis), Fokker–Planck reductions, and other techniques from stochastic calculus developed previously for PDEs. For a recent perspective on this approach the book by Bressloff is a highly valuable resource [16], as well as the paper by Inglis and MacLaurin [17], which presents a general framework in which to rigorously study the effect of spatio-temporal noise on travelling wave fronts. Indeed there is now a quite elegant body of rigorous theory growing up around neural field models with multiplicative stochastic forcing, as exemplified in the paper by Krüger and Stannat [18] using multiscale analysis, which moves beyond formal perturbation methods, to understand front propagation in particular. However, the original work of Wilson and Cowan suggests that another, perhaps more natural, way to introduce stochasticity into neural field models is by treating some of the system parameters as random variables. Indeed, threshold noise in a linear integrate-and-fire model is able to fit real firing patterns observed in the sensory periphery [19]. The simplicity of such models is also appealing from a theoretical perspective, and for a threshold described by an Ornstein–Uhlenbeck process it has recently been shown that analytical (and non-perturbative) expressions for the first-passage time distribution can be obtained [20].

To appreciate the original idea of Wilson and Cowan that threshold noise in switching networks can give rise to a probabilistic interpretation of network dynamics in terms of a smooth firing-rate function it is enough to consider a simple discrete time model for the evolution of neural activity $x_{i}(t)$, $i=1,\ldots, N$, in a network with connections $w_{ij}$:

1 $$ x_{i}(t+1) = H \biggl( \sum_{j} w_{ij} x_{j}(t) - h \biggr) . $$

Here *H* is a Heaviside step function with threshold *h*. If we now associate a threshold $h_{i}$ with each individual node and treat it as a random variable drawn from a normalised stationary probability distribution $\phi(h_{i})$ at each time step then we can take the ensemble average of the above and find

2 $$ x_{i}(t+1) = f \biggl( \sum_{j} w_{ij} x_{j}(t) \biggr) , $$

where $f(u) = \int_{-\infty}^{\infty}H(u-h) \phi(h) \,\mathrm {d}h$. Thus we obtain a smooth nonlinear deterministic model describing the average behaviour of a set of switch like elements with random thresholds, with the link between the two determined by the relationship $f'=\phi$. Since *ϕ* is a probability distribution, this relationship immediately implies a monotonically increasing firing-rate function. Given a realisation of the thresholds $h_{i}$ at some time *t*, it is of interest to ask how the spatial covariance structure of these random thresholds affects network dynamics. This is precisely the question we wish to address in this paper for continuum models of Wilson–Cowan type, in which the random firing threshold is now described as spatially continuous quenched disorder. Although we will restrict our attention to a Gaussian covariance function, we shall consider a broad class of stationary distributions, and present practical techniques from applied mathematics and statistics for working with non-Gaussian distributions. Moreover, by working with the Heaviside choice, as in (1), we will be able to build on the interface approach of Amari [21] to obtain explicit results for travelling fronts and bumps, and their dependence on the threshold noise structure.

In Sect. 2 we introduce our neural field model of choice, as well as the form of the stochastic threshold, namely its steady state distribution and spatial covariance structure. In Sect. 3 we show that, for a given realisation of the threshold, we may use the Amari interface approach to determine the instantaneous speed of a travelling front. We further show how to calculate the effects of the quenched spatial disorder arising from the noisy threshold using a perturbative approach, valid for small deviations of the threshold from its average value. We extend the approach for fronts to tackle stationary bumps in Sect. 4, where we also show how to determine the linear stability of localised solutions. This leads to a prediction that noise can induce multiple stable bumps, which we confirm numerically. Indeed throughout the paper we use direct numerical simulations to illustrate the accuracy of all theoretical predictions. Finally in Sect. 5 we discuss natural extensions of the work in this paper.

## The Model

For mathematical convenience it is often easier to work with spatially continuous models rather than lattice models of the type described by (1). We consider a neural field $u=u(x,t) \in \mathbb {R}$, $x \in[0,L]$, $t \in \mathbb {R}^{+}$, whose dynamics is given by

3 $$ \frac{\partial u}{\partial t} = -u + \int_{0}^{L} w\bigl(\vert x-y\vert \bigr) H \bigl(u(y,t)-h(y)\bigr) \,\mathrm {d}y . $$

The kernel *w* represents the anatomical connectivity, and we have chosen to include the nonlinearity within the spatial integration, though activity based models with the nonlinearity outside the spatial integration may also be considered with the techniques described below (and are qualitatively similar in their behaviour). As it stands the model given by (3) is a standard Amari neural field model for the choice that *h*, the firing threshold, is a constant function. In this case the model is well known to support travelling waves, including fronts [22] and localised bump states in systems with a mixture of excitation and inhibition. For a review of such behaviour see [23], and for a recent overview of neural field modelling in general see [3].

In this paper we shall consider the case that *h* is a spatially random function. Given the wealth of mathematical knowledge for Gaussian disorder it would be highly convenient to make this choice for the threshold. However, this is a non-physiological convenience that we would prefer to avoid. Indeed it is very natural to expect threshold noise to be bounded and unlikely to be best described by a symmetric distribution. As such we will consider both Gaussian and non-Gaussian disorder and in particular skewed exponential distributions and distributions with compact support. We shall explicitly model the random firing threshold $h(x)$ as

4 $$ h(x)=h_{0}+\epsilon g(x) , $$

where $h_{0}>0$ corresponds to the mean of the threshold, and $g(x)$ denotes the quenched disorder with symmetric, bounded and non-negative spatial covariance function $C(x,y)$. We shall fix this to be a Gaussian shape such that $C(x,y)=C(\vert x-y\vert )$, with

5 $$ C(x)=\sigma^{2} \exp \biggl( -\pi\frac{x^{2}}{\kappa^{2}} \biggr) . $$

Here *κ* is the correlation length of the quenched disorder. Note that the variance of the threshold is given by $\epsilon^{2} \sigma^{2}$. There exists a sequence of non-negative real numbers, $\lambda_{m}$, $m \geq1$, which are eigenvalues of the covariance operator, associated with a sequence of eigenfunctions, $e_{m}$, $m \geq1$, according to

6 $$ \int_{0}^{L} C(x,y) e_{m}(y) \,\mathrm {d}y = \lambda_{m} e_{m}(x), $$

that form a complete orthonormal basis so that we may represent $g(x)$ by its Karhunen–Loève decomposition [24–26]

7 $$ g(x)=\sum_{m=1}^{\infty}\sqrt{ \lambda_{m}} \alpha_{m} e_{m}(x) . $$

Here the $\alpha_{m}$ are uncorrelated random variables with zero mean and unit variance, i.e. $\mathbb{E}(\alpha_{m})=0$ and $\mathbb {E}(\alpha _{m} \alpha_{n})=\delta_{mn}$. The properties of the $\alpha_{m}$ ensure that the Karhunen–Loève representation captures the first and second moment of $g(x)$ exactly. The latter result follows from the fact that

8 $$\begin{aligned} C(x,y) & = \mathbb{E} \bigl( \bigl[g(x)- \mathbb{E} (g)\bigr] \bigl[g(y)- \mathbb{E} (g)\bigr] \bigr) = \mathbb{E} \bigl( g(x) g(y) \bigr) \\ &= \sum_{m,n} \sqrt{\lambda_{m} \lambda_{n}} e_{m}(x) e_{n}(y) \mathbb{E} ( \alpha_{m} \alpha_{n}) = \sum_{m} \lambda_{m} e_{m}(x) e_{m}(y) , \end{aligned}$$

so that $C(x,y)$ has the expected spectral representation. When the correlation length *κ* is much smaller than the domain size *L*, the Karhunen–Loève decomposition of $g(x)$ for the Gaussian covariance function (5) with *periodic boundary conditions* can be very well approximated by [25]

9 $$ g(x)=\sum_{m=0}^{\infty}\beta_{m} \sqrt{\lambda_{m}}e_{m}^{(1)}(x)+\sum _{m=1}^{\infty}\gamma_{m}\sqrt{ \lambda_{m}}e_{m}^{(2)}(x) . $$

Here we have split the eigenfunctions $e_{m}(x)$ in (7) into two sets $e_{m}^{1}(x)$ and $e_{m}^{2}(x)$, which read

10 $$ e_{m}^{(1)}(x)=\sqrt{\frac{2}{L}}\cos{( \omega_{m} x)} ,\qquad e_{m}^{(2)}(x)=\sqrt{ \frac{2}{L}}\sin{(\omega_{m} x)} ,\quad m\geq1 , $$

with $\lambda_{m}=\sigma^{2} \kappa\exp{[-\omega_{m}^{2} \kappa^{2}/(4 \pi)]}$, $\omega_{m}=2\pi m/L$ and $e_{0}^{(1)}=\sqrt{1/L}$. Note that we have

11 $$ \int_{0}^{L} e_{m}^{(1)}(x)e_{n}^{(2)}(x) \,\mathrm {d}x=0 ,\qquad \int_{0}^{L} e_{m}^{(i)}(x)e_{n}^{(i)}(x) \,\mathrm {d}x=\delta_{mn} ,\quad i=1,2 , $$

for any *n*, *m*. To complete the model setup we need to specify the random coefficients $\beta_{m}$ and $\gamma_{m}$. They are determined by the local distribution $\phi(g)$ of the quenched disorder $g(x)$. If $\phi(g)$ is Gaussian, it suffices to choose the $\beta_{m}$ and $\gamma _{m}$ as uncorrelated univariate Gaussian random variables, namely $\mathbb{E} (\beta_{m})=0=\mathbb{E} (\gamma_{m})$ and $\mathbb{E} (\beta_{m} \beta_{n})=\delta_{mn}=\mathbb{E} (\gamma_{m} \gamma_{n})$. Indeed there is a large variety of methods to simulate Gaussian disorder including autoregressive-moving-averages [27], circular embedding [28] spectral representations [29] or the Karhunen–Loève decomposition [24–26]. However, if $\phi(g)$ is non-Gaussian, then $\beta_{m}$ and $\gamma_{m}$ are not described by a scaled version of $\phi(g)$. Thus we require suitable techniques to generate non-Gaussian disorder.

**Fig. 1:**
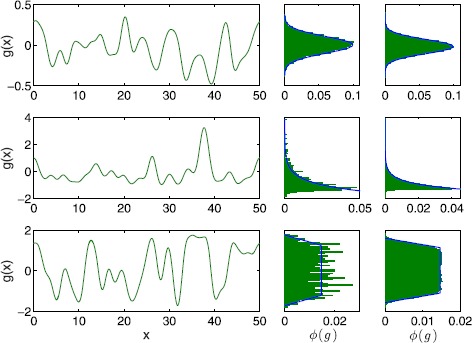
Random thresholds. Realisations of the random threshold (*left*) for a Gaussian (*top*), shifted exponential (*middle*) and bump (*bottom*) distribution. *The middle column* shows the probability distribution of the threshold on *the left*. *The right column* depicts the probability distribution of the threshold obtained from 1000 realisations. Equation (9) was truncated after 50 (*top*), 32 (*middle*) and 64 (*bottom*) terms per sum, respectively. Parameter values are $L=50$, $\kappa=3$ and $\sigma=0.2$ (*top*), $\lambda=1$, $\mu=-1$ (*middle*) and $A=\sqrt{2}$, $B=2$, $\alpha=0.5$ (*bottom*)

Compared to Gaussian statistics, there are only a few methods for simulating non-Gaussian disorder. Amongst them, translation processes, in which a suitably chosen Gaussian model is non-linearly mapped to the desired non-Gaussian disorder, and a Karhunen–Loève decomposition feature most prominently [30, 31]. We have chosen to employ a Karhunen–Loève decomposition as it is more robust [32], which means that we may use the same technique for both Gaussian and non-Gaussian disorder. We implement the method developed in [30] and provide details of the algorithm in Appendix A, though the main idea is as follows. As a starting point choose uncorrelated $\beta_{m}$ and $\gamma _{m}$ from the probability distribution $\phi(g)$ and generate a large number of samples of the quenched disorder $g(x)$ according to (7). Since the $\beta_{m}$ and $\gamma_{m}$ are uncorrelated $g(x)$ possesses the prescribed covariance function $C(x,y)$. However, the probability distribution of $g(x)$ differs from $\phi(g)$. This can be corrected by determining a new set of the $\beta_{m}$ and $\gamma_{m}$, but these $\beta_{m}$ and $\gamma_{m}$ are now correlated. It is then possible to decorrelate the $\beta_{m}$ and $\gamma_{m}$ without changing their statistics, which in turn ensures that the statistics of $g(x)$ still comply with $\phi(g)$. Overall, the method in [30] provides an iterative scheme such that the probability distribution of $g(x)$ converges towards the prescribed distribution $\phi(g)$, while keeping the chosen covariance function exact in every iteration step. In Fig. 1 we show the three types of distribution that we use to realise threshold values. These are (i) a Gaussian distribution, (ii) a highly skewed shifted exponential distribution, and (iii) a piecewise linear distribution with compact support. The precise mathematical form for each of these is given in Appendix B.

## Travelling Fronts

As mentioned in Sect. 1 much is now known about the effects of random forcing on neural field models. As regards travelling fronts the work of Bressloff and Webber [9] has shown that this can result in ‘fast’ perturbations of the front shape as well as a ‘slow’ horizontal displacement of the wave profile from its uniformly translating position. A separation of time-scales method is thus ideally suited to analysing this phenomenon, though we also note that more numerical techniques based upon *stochastic freezing* [33] could also be utilised. In this section we will explore the effects of quenched or ‘frozen’ threshold noise on the properties of a travelling wave, and in particular its speed.

For a symmetric choice of synaptic kernel $w(x)=w(\vert x\vert )$, which decays exponentially, the one-dimensional model (3) with a constant threshold is known to support a travelling front solution [22, 23] that connects a high activity state to a low activity state. In this case it is natural to define a pattern boundary as the interface between these two states. One way to distinguish between the high and the low activity state is by determining whether *u* is above or below the firing threshold. When denoting the position of the moving interface by $x_{0}(t)$, the above notion leads us to the defining equation

12 $$ u\bigl(x_{0}(t),t\bigr) = h\bigl(x_{0}(t)\bigr) . $$

**Fig. 2:**
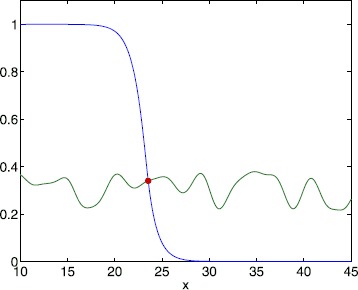
Travelling front. Instance of a travelling front (*blue*) for the bump distribution. The threshold is shown in *green*. *The red dot* indicates the position $x_{0}(t)$ where $u(x_{0}(t),t)=h(x_{0}(t))$. Equation (9) was truncated after 64 terms per sum. Parameter values are $A=\sqrt{2}$, $B=2$, $\alpha=0.5$, $L=50$, $\kappa=3$, $\epsilon=0.05$ and $h_{0}=0.3$

Here, we are assuming that there is only one point on the interface as illustrated in Fig. 2, though in principle we could consider a set of points. For the choice (5) we see that $C(x)$ is differentiable at $x=0$, which means that the random threshold is differentiable in the mean square sense. The differentiation of (12) gives an exact expression for the velocity of the interface *c* in the form

13 $$ c\equiv\frac{\mathrm {d}x_{0}}{\mathrm {d}t} = \frac{u_{t}}{h_{x}-u_{x}} \bigg\vert _{x=x_{0}(t)} , $$

which modestly extends the original approach of Amari [21] with the inclusion of the term for $h_{x}$. We can now describe the properties of a front solution solely in terms of the behaviour at the front edge that separates high activity from low, as described in [34, 35]. To see this, let us consider a right moving front for which $u(x,t) > h(x)$ for $x < x_{0}(t)$ and $u(x,t) \leq h(x)$ for $x \geq x_{0}(t)$. Then we solve (3), dropping transients, to obtain

14 $$ u(x,t) = \int_{0}^{t} \mathrm {e}^{-(t-s)} \psi(x,s) \,\mathrm {d}s,\qquad \psi(x,t) = \int _{-\infty}^{x_{0}(t)} w(x-y) \,\mathrm {d}y . $$

Hence,

15 $$ u\bigl(x_{0}(t),t\bigr) = \int_{0}^{t} \,\mathrm {d}s \mathrm {e}^{-(t-s)} \int_{x_{0}(t)-x_{0}(s)}^{\infty}\,\mathrm {d}y w(y) . $$

For simplicity we make the choice $w(x)=\exp(-\vert x\vert )/2$ so that from (14) we find by differentiation with respect to *x* that for a right moving wave (for large *t*)

16 $$ u\bigl(x_{0}(t),t\bigr) = - u_{x} \vert _{x=x_{0}(t)} . $$

By noting that

17 $$ u_{t} \vert _{x=x_{0}(t)}=-h\bigl(x_{0}(t) \bigr)+ \int_{-\infty}^{x_{0}(t)} w\bigl(x_{0}(t)-y\bigr) \, \mathrm {d}y=-h\bigl(x_{0}(t)\bigr)+\frac{1}{2} , $$

and inserting (16) and (17) into (13), we find the wave speed $c_{+}$ of a right moving wave

18 $$ c_{+}=\frac{1-2 h(x_{0}(t))}{2h_{x}(x_{0}(t))+2h(x_{0}(t))} . $$

When we repeat the above derivation for a left moving wave, the wave speed $c_{-}$ is given by

19 $$ c_{-}=\frac{1-2 h(x_{0}(t))}{2h_{x}(x_{0}(t))+2-2h(x_{0}(t))} , $$

where we used

20 $$ u\bigl(x_{0}(t),t\bigr)=1+ u_{x} \vert _{x=x_{0}(t)} . $$

Note that in the case of a constant threshold with $h(x)=h_{0}$ we obtain $c_{+} = {(1-2 h_{0})}/(2h_{0})$, for $h_{0} < 1/2$, and $c_{-} = (1-2 h_{0})/(2(1-h_{0}))$, for $1/2 < h_{0} < 1$, which recovers a previous result, as discussed in [16]. If the front is moving to the right we have an exact expression for the speed (see also [36]):

21 $$ c(x)=\frac{1-2h(x)}{2h(x)+2h_{x}(x)} . $$

**Fig. 3:**
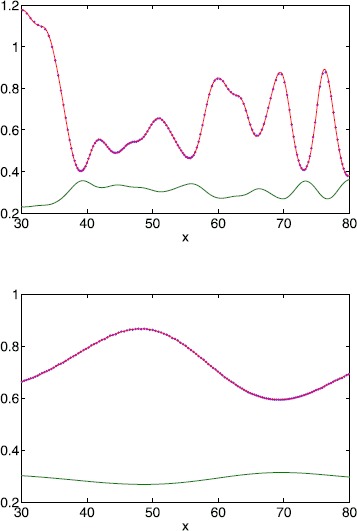
Instantaneous speed of a travelling front for a Gaussian threshold distribution. Measured front speed for a Gaussian threshold distribution, extracted from a simulation of (3) (*blue*); theoretical value from (21) (*red*); and the threshold (4) (*green*), for $\kappa =5$ (*top*) and $\kappa =30$ (*bottom*). Equation (9) was truncated after 50 terms per sum. Other parameter values are $\sigma^{2}=1/\kappa$, $h_{0}=0.3$, $\epsilon =0.01$, $L=100$

**Fig. 4:**
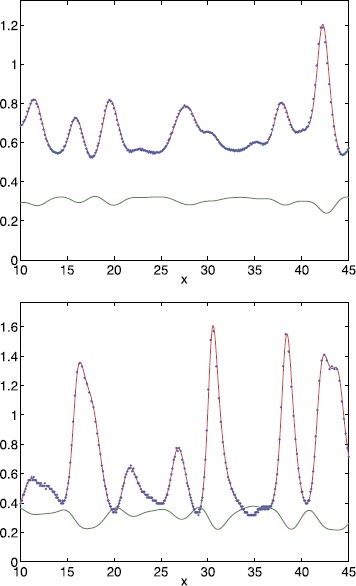
Instantaneous speed of a travelling front for non-Gaussian threshold distributions. Measured front speed, extracted from a simulation of (3) (*blue*); theoretical value from (21) (*red*); and the threshold (4) (*green*) for a shifted exponential distribution (*top*) and a bump distribution (*bottom*) for $\kappa=3$, $h_{0}=0.3$ and $L=50$. Equation (9) was truncated after 32 (*top*) and 64 (*bottom*) terms per sum, respectively. Other parameter values are $\lambda=1$, $\mu=-1$, $\epsilon=-0.03$ (*top*) and $A=\sqrt {2}$, $B=2$, $\alpha=0.5$, $\epsilon=0.05$ (*bottom*)

Examples of this relationship are shown in Figs. 3 and 4 where we plot both $c(x)$ and the instantaneous front velocity extracted from a numerical simulation of (3). Figure 3 depicts results when the local probability distribution is a Gaussian for two different values of the correlation length *κ*, while Fig. 4 illustrates travelling fronts for thresholds that are locally distributed as a skewed exponential and a bump. We see excellent agreement between the numerical values and the expression (21).

### Perturbative Calculation of Wave Speed

We can also perturbatively calculate the effects of threshold disorder on the speed of a travelling front. Substituting (4) into (21) and taking $\epsilon \ll1$ we find that

22 $$\begin{aligned} c(x) \simeq{}&\frac{1-2h_{0}}{2h_{0}} -\frac{\epsilon}{2 h_{0}} \biggl[\frac {g(x)}{h_{0}}+ \frac{1-2 h_{0}}{h_{0}} g'(x) \biggr] \\ &{}+\frac{\epsilon^{2}}{2h_{0}^{2}} \biggl[\frac {g(x)^{2}+2g(x)g'(x)+g'(x)^{2}}{h_{0}}-2g(x)g'(x)-2g'(x)^{2} \biggr] , \end{aligned}$$

where the prime denotes differentiation. We will now take the spatial and ensemble average of (22). It is convenient to introduce an angle bracket notation to denote spatial averaging according to $\langle\cdot\rangle\equiv\int_{0}^{L} \cdot \mathrm {d}x/L$. We find from (9) that $\langle g \rangle= \beta_{0} \sqrt {\lambda _{0}/L}$ (since only the constant eigenfunction $e_{0}^{(1)}(x)$ contributes to the integral and all other terms in (9) integrate to zero because of periodicity) and $\langle g' \rangle= 0$ (because of periodicity). Hence, the spatial average of *c* takes the compact form

23 $$ \langle c \rangle\simeq\frac{1-2h_{0}}{2h_{0}}-\frac{\epsilon\langle g \rangle}{2 h_{0}^{2}} +\frac{\epsilon^{2}}{2h_{0}^{3}} \bigl[ \bigl\langle g^{2} \bigr\rangle +(1-2h_{0}) \bigl\langle g^{\prime 2} \bigr\rangle \bigr] , $$

where $L \langle g^{2} \rangle= \sum_{m=0}^{\infty}\beta_{m}^{2}\lambda _{m}+\sum_{m=1}^{\infty}\gamma_{m}^{2}\lambda_{m}$ and $L\langle g^{\prime2 }\rangle= \sum_{m=1}^{\infty}\beta_{m}^{2}\lambda_{m}\omega_{m}^{2}+ \sum_{m=1}^{\infty}\gamma _{m}^{2}\lambda_{m} \omega_{m}^{2}$. Now taking expectations over the $\beta_{m}$ and $\gamma_{m}$ we obtain $\mathbb{E} ( \langle g \rangle )=0$, $L \mathbb{E} (\langle g^{2} )\rangle= \lambda_{0}+2\sum_{m=1}^{\infty}\lambda_{m}$ and $L \mathbb{E} (\langle g^{\prime2}) \rangle= 2\sum_{m=1}^{\infty}\lambda_{m} \omega_{m}^{2}$. Hence, we may construct $\overline{c} = \mathbb{E} ( \langle c \rangle )$ as

24 $$ \overline{c} \simeq\frac{1-2h_{0}}{2h_{0}}+\frac{\epsilon^{2}}{h_{0}^{3} L} \Biggl[ \frac{\lambda_{0}}{2}+\sum_{m=1}^{\infty}\lambda_{m} + (1-2h_{0} )\sum_{m=1}^{\infty}\lambda_{m}\omega_{m}^{2} \Biggr] . $$

This expression gives the lowest order correction term to the expression for speed (for a right moving wave) when the threshold is constant and takes the value $h_{0}$. The term in square brackets in (24) is positive, and thus spatial disorder will always increase the average speed. This term also increases as the correlation length decreases since the $\lambda_{m}$ decay more slowly for smaller correlation lengths. Note, however, that the correction term remains uniformly small since $\lambda_{m} \sim\kappa$ for all *m* when $\kappa\ll1$.

**Fig. 5:**
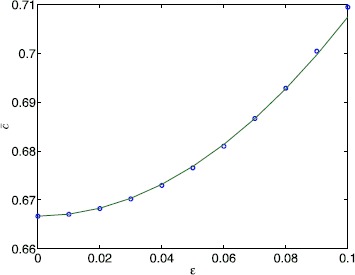
Mean speed versus *ϵ* for a Gaussian threshold distribution. *c̅* as a function of *ϵ* for $h_{0}=0.3$. *Solid curve*: (24); *circles*: from averaging (21) over 1000 realisations per point. Equation (9) was truncated after 50 terms per sum. Other parameter values are $\kappa=5$, $\sigma^{2}=0.2$, $L=100$

**Fig. 6:**
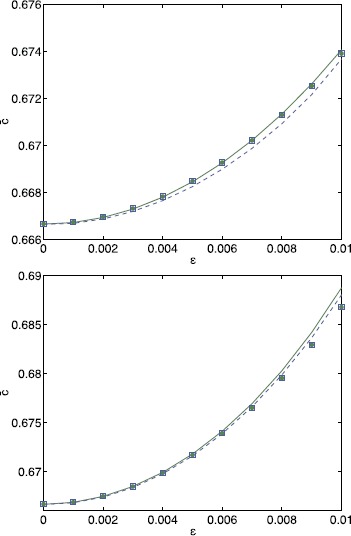
Mean speed versus *ϵ* for non-Gaussian threshold distributions. *c̅* as a function of *ϵ* for $\kappa=0.5$, $h_{0}=0.3$, $L=100$ for the exponential distribution (*top*) and the bump distribution (*bottom*). In *each panel* results for the non-Gaussian distribution (*dashed blue*) are compared to those for a Gaussian distribution (*solid green*) with the same variance. *Solid/dashed curves*: (24); *blue squares* (non-Gaussian)/*green circles* (Gaussian): from averaging (21) over 1000 realisations per point. Equation (9) was truncated after 250 terms per sum. Other parameter values are $\lambda=1.66$, $\mu=-0.6$ (*top*) and $A=\sqrt{2}$, $B=2$, $\alpha=0.5$ (*bottom*)

Figure 5 shows *c̅* as a function of *ϵ* for a Gaussian distribution of threshold values, as well as results from directly averaging realisations of (21). Here the $\beta_{m}$ and $\gamma_{m}$ are randomly chosen from the unit normal distribution. We obtain identical results when these are uniformly distributed on $[-\sqrt{3},\sqrt{3}]$ (i.e. with mean zero and variance 1). The difference between using bounded distributions for the $\beta_{m}$ versus unbounded is that the maximum value of *h* is then bounded/unbounded. The results based on (24) are almost identical to those obtained from (21) for small values of *ϵ*, while minor deviations appear as we increase *ϵ*. In Fig. 6 we plot *c̅* as a function of *ϵ* for the shifted exponential and bump distribution. In addition, we show results for a Gaussian distribution with the same variance. We again observe very good agreement between the small noise expansion (24) and averaging (21) for small values of *ϵ*. In addition, the curves for the Gaussian threshold and for the non-Gaussian thresholds obtained from averaging (21) almost agree, while the expansion (24) yields identical results for both kinds of threshold noise. The latter is a direct consequence of the bi-orthogonality of the Karhunen–Loève expansion. Equation (24) only depends on the eigenvalues of the covariance function and not on the properties of the local distributions.

## Stationary Bumps

Neural fields of Amari type are known to support spatially localised stationary bump patterns when the anatomical connectivity function has a Mexican-hat shape. In a one dimensional spatial model, and in the absence of noise, it is known that pairs of bumps exist for some sufficiently low value of a constant threshold and that only the wider of the two is stable [21]. For random forcing it is possible to observe noise-induced drifting activity of bump attractors, which can be described by an effective diffusion coefficient (using a small noise expansion) [11] or by an anomalous sub-diffusive process in the presence of long-range spatio-temporal correlations [37]. However, it is also known that spatial disorder can act to *pin* states to certain network locations by breaking the (continuous) translation symmetry of the system, as described in [38] for neural field models with heterogeneous anatomical connectivity patterns. It is the latter phenomenon that we are interested in here, especially since for a disordered threshold that breaks translational symmetry, it is not a priori obvious how many bump solutions exist and what their stability properties are.

A one-bump solution $q(x)$ is characterised by a width Δ, such that for two values $x_{1}$ and $x_{2}$ with $\Delta=x_{2}-x_{1}$ we have $q(x)\geq h(x)$ for $x_{1} \leq x \leq x_{2}$. Using (3) a one-bump solution therefore satisfies

25 $$ q(x) = \int_{x_{1}-x}^{x_{2}-x} w(y) \,\mathrm {d}y . $$

Note that $h(x_{1})=q(x_{1})=h(x_{2})=q(x_{2})=U(\Delta)$ with $U(\Delta)$ given by

26 $$ U(\Delta) = \int_{0}^{\Delta}w\bigl(\vert y\vert \bigr) \,\mathrm {d}y . $$

We can determine the linear stability of bumps by studying the (linearised) evolution of a perturbation $v(x) \mathrm {e}^{\lambda t}$ around the stationary bump $q(x)$. We hence find from (3)

27 $$\begin{aligned} (1+\lambda) v(x) &= \int_{\mathbb {R}} w(x-y) \delta\bigl(q(y)-h(y)\bigr) v(y)\, \mathrm {d}y \end{aligned}$$

28 $$\begin{aligned} &= \frac{w(x-x_{1})}{\vert Q'(x_{1})\vert } v(x_{1}) + \frac{w(x-x_{2})}{\vert Q'(x_{2})\vert } v(x_{2}), \end{aligned}$$

where $Q(x)=q(x)-h(x)$. Demanding that the perturbations at $x_{1,2}$ be non-trivial yields the spectral equation $\det(\mathcal{A}-(1+\lambda)I_{2})=0$, where $I_{2}$ is the identity matrix in $\mathbb {R}^{2 \times2}$ and

29 $$ \mathcal{A} = \begin{bmatrix} \frac{w(0)}{\vert Q'(x_{1})\vert } & \frac{w(\Delta)}{\vert Q'(x_{2})\vert } \\ \frac{w(\Delta)}{\vert Q'(x_{1})\vert } & \frac{w(0)}{\vert Q'(x_{2})\vert } \end{bmatrix} . $$

The eigenvalues are then given by $\lambda=\lambda_{\pm}$:

30 $$ 1+\lambda_{\pm}= \frac{1}{2} \bigl\{ \operatorname {Tr}\mathcal{A} \pm \sqrt {(\operatorname {Tr}\mathcal{A})^{2} - 4 \det\mathcal{A}} \bigr\} . $$

Note further that for $h'(x)=0$ we have $\vert Q'(x_{1})\vert =\vert Q'(x_{2})\vert = \vert w(0)-w(\Delta)\vert $ and therefore $\lambda_{-}=0$ as expected from translation invariance.

### Simple Heterogeneity

We first consider a simple form of heterogeneity to present the ideas, and then move to more general heterogeneity. Suppose $w(x)=\mathrm{e}^{-\alpha(1-\cos{x})}-B\mathrm{e}^{-\beta(1-\cos{x})}$ and $h(x)=h_{0}+\epsilon\cos{x}$, and the domain is $[0,2\pi]$. Then we have bumps which have their maximum at either 0 or *π*. Suppose the maximum is at 0 and $x_{1}=-a$ for $0< a<\pi$. Since we need $h(x_{1})=h(x_{2})$ and the threshold is symmetric around 0, we immediately arrive at $x_{2}=a$. To determine *a* we require $U(\Delta)=U(2a)=h(a)$ i.e.

31 $$ \int_{0}^{2a}\mathrm{e}^{-\alpha(1-\cos{x})}-B\mathrm{e}^{-\beta(1-\cos{x})} \,\mathrm {d}x=h_{0}+\epsilon\cos{a} . $$

**Fig. 7:**
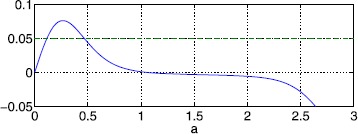
Widths of stationary bumps. The left hand side of (31) (*solid*) and the right hand side (*dashed*), with $\alpha=5$, $B=0.76$, $\beta=3$, $h_{0}=0.05$ and $\epsilon=0$

**Fig. 8:**
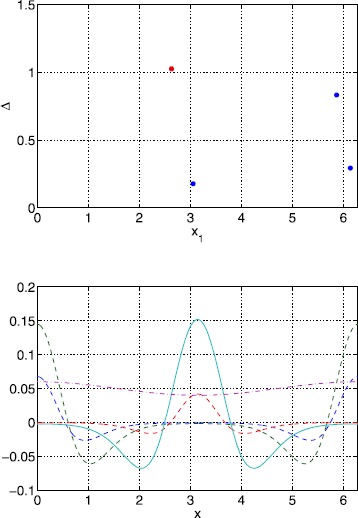
Bump widths and profiles for a spatially heterogeneous threshold. *Top*: solutions of (32)–(33), where $\Delta =x_{2}-x_{1}$, for $h(x)=0.05+0.01\cos{x}$. Only *the red* solution is stable. *Bottom*: bump profiles for the solutions in *the upper panel*, and threshold $h(x)$ (*dash-dotted*). *The solid* bump is stable, all others (*dashed*) are unstable. Parameter values are $\alpha=5$, $B=0.76$ and $\beta=3$

Choosing $\alpha=5$, $B=0.76$, $\beta=3$, $h_{0}=0.05$ and $\epsilon=0$ we have two bump widths, as shown in Fig. 7 ($a=\Delta/2$). We find that the larger root is stable and the other is unstable, and both have a zero eigenvalue as expected. Increasing *ϵ* from zero breaks the translational invariance of the system and we obtain 4 bumps for $\epsilon=0.01$ as shown in Fig. 8: the two that exist for the homogeneous case ($\epsilon =0$), now centred around $x=\pi$, and similarly two centred around $x=0$. (We no longer restrict to $x_{1}<0$.)

### General Heterogeneity

We keep $w(x)=\mathrm{e}^{-\alpha(1-\cos{x})}-B\mathrm{e}^{-\beta(1-\cos{x})}$ and now consider a general $h(x)$ without symmetries. The problem of the existence of a bump is this: given $h(x)$ and a value of $x_{1}$, find a value of $x_{2}$ such that $h(x_{2})=h(x_{1})$, and $U(\Delta)=U(x_{2}-x_{1})=h(x_{1})$, where $U(x)$ is given by (26). This will not happen generically but only at isolated points in the $(x_{1},x_{2})$ plane. Thus we need to solve the simultaneous equations

32 $$\begin{aligned} h(x_{2}) & =h(x_{1}) , \end{aligned}$$

33 $$\begin{aligned} U(x_{2}-x_{1}) & =h(x_{1}) , \end{aligned}$$

and we only search for solutions which satisfy

34 $$ 0< x_{1}< 2\pi\quad \mbox{and}\quad x_{1}< x_{2}< x_{1}+2 \pi. $$

We now apply this general concept to the stochastic threshold (9). For each realisation and set of parameter values we use Newton’s method with 1000 randomly chosen initial values which satisfy (34). Out of these 1000 initial values, the number of distinct solutions of (32)–(33) that satisfy (34) is recorded, and their stability is determined as described above. We then check these solutions to verify that $q(x)>h(x)$ only for $x_{1}< x< x_{2}$. Any that do not satisfy this inequality are discarded.

**Fig. 9:**
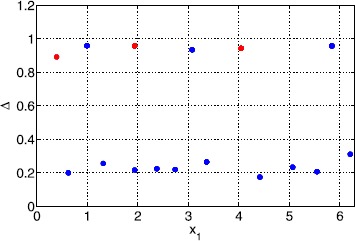
Bump widths for a Gaussian threshold distribution. Typical sets of solutions of (32)–(33) for a Gaussian threshold distribution with $\kappa=1$, $\sigma=1$ and $\epsilon=0.01$. Only those with *red dots* are stable. Other parameter values are $\sigma=1$, $\alpha=5$, $B=0.76$, $\beta=3$ and $h_{0}=0.05$

**Fig. 10:**
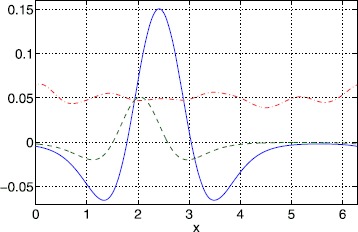
Bumps for a Gaussian threshold distribution. Stable (*solid*) and unstable (*dashed*) solutions corresponding to the two points in Fig. 9 with $x_{1}$ slightly less than 2. The threshold is shown *dash-dotted*. Parameter values are $\kappa=1$, $\epsilon=0.01$, $\sigma=1$, $\alpha=5$, $B=0.76$, $\beta=3$ and $h_{0}=0.05$

**Fig. 11:**
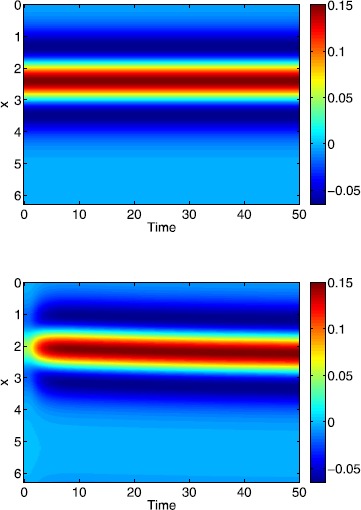
Space-time plots of bumps for a Gaussian threshold distribution. Simulations of (3) using as initial conditions the two different solutions shown in Fig. 10. *Top*: stable; *bottom*: unstable. Parameter values are $\kappa=1$, $\epsilon=0.01$, $\sigma=1$, $\alpha=5$, $B=0.76$, $\beta=3$ and $h_{0}=0.05$

Typical results for a Gaussian threshold are depicted in Fig. 9 for $\kappa=1$ and $\epsilon=0.01$. We see that width values ($\Delta\equiv x_{2}-x_{1}$) are clustered around two values (those corresponding to the homogeneous system) and that the only stable ones are those with large widths (inherited from the homogeneous case). Two example bumps, one stable and the other unstable, from Fig. 9 are shown in Fig. 10. Space-time simulations of (3) using these two bumps as initial conditions are shown in Fig. 11. The stable bump is stationary, as expected, while the solution starting close to (but not exactly at) the unstable bump rapidly increases in size and then slowly moves towards a stable solution.

**Fig. 12:**
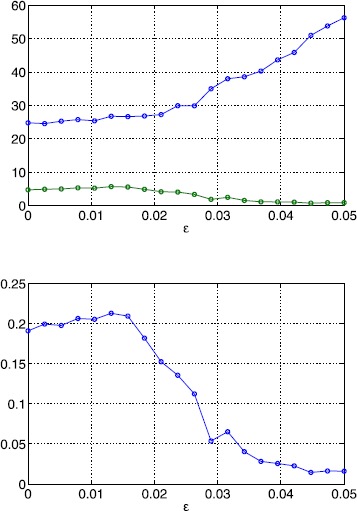
Bump solutions as a function of *ϵ* for a Gaussian threshold distribution. *Top*: average number of total solutions (*blue*) and stable solutions (*green*) of (32)–(33) for a Gaussian threshold distribution. *Bottom*: fraction of solutions which are stable. Parameter values are $\alpha=5$, $B=0.76$, $\beta=3$, $h_{0}=0.05$, $\kappa=0.5$ and $\sigma^{2}=4$

**Fig. 13:**
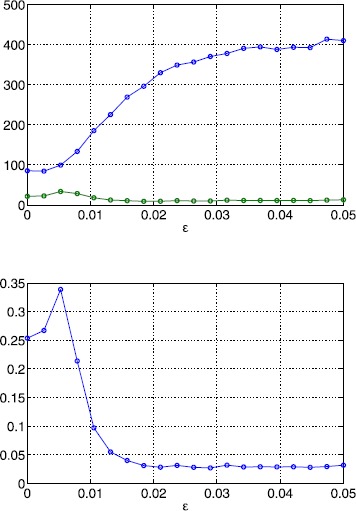
Bump solutions as a function of *ϵ* for a Gaussian threshold distribution. *Top*: average number of total solutions (*blue*) and stable solutions (*green*) of (32)–(33) for a Gaussian threshold distribution. *Bottom*: fraction of solutions which are stable. Parameter values are $\alpha=5$, $B=0.76$, $\beta=3$, $h_{0}=0.05$, $\kappa=0.1$ and $\sigma^{2}=10$

**Fig. 14:**
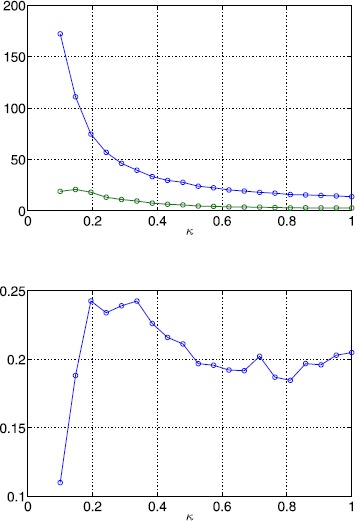
Bump solutions as a function of *κ* for a Gaussian threshold distribution *Top*: average number of total solutions (*blue*) and stable solutions (*green*) of (32)–(33) for a Gaussian threshold distribution. *Bottom*: fraction of solutions which are stable. Parameter values are $\alpha=5$, $B=0.76$, $\beta=3$, $h_{0}=0.05$, $\epsilon=0.01$ and $\sigma^{2}=1/\kappa$

In Fig. 12 we show the number of solutions of (32)–(33) as well as the number and fraction of stable solutions as a function of *ϵ*. While the number of solutions exhibits an increasing trend, the number of stable solutions decreases, leading to an overall decrease in the fraction of stable solutions. When lowering the correlation length five times (Fig. 13), we observe a similar behaviour. Note, however, that the number of solutions has increased significantly. When we fix *ϵ* and vary *κ* the number of solutions decays quickly as shown in Fig. 14. At the same time, the number of stable solutions remains almost constant, resulting in a strong increase of the fraction of stable solutions. Overall, we find that varying the amplitude of the threshold heterogeneity by changing *ϵ* more strongly affects solution numbers when *κ* is small, and that the value of *κ* has a significant effect on how many fixed points exist (and a lesser effect on the number of those which are stable).

**Fig. 15:**
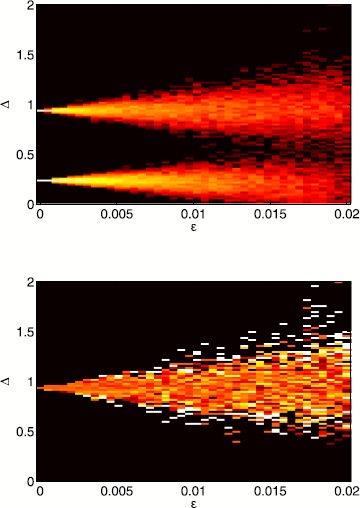
Probability distributions of bump widths for a Gaussian threshold distribution. *Top*: log of the probability density of Δ values for a Gaussian threshold distribution. (*White* is high, *black* low.) *Bottom*: fraction of solutions which are stable. ($\mathit {Black}=0$, $\mathit{white}=1$.) Parameter values are $\alpha=5$, $B=0.76$, $\beta=3$, $h_{0}=0.05$, $\kappa=0.5$ and $\sigma^{2}=4$

A more detailed view on bump solutions with a Gaussian threshold is presented in Fig. 15, where we show the distribution of Δ values as a function of *ϵ* and the proportion which are stable. For $\epsilon=0$ the probability distribution consists of two delta functions at the stable and unstable bump, respectively. As *ϵ* increases two branches of solutions emerge from the solutions in the homogeneous case, which widen for larger values of *ϵ*. Note that as in the homogeneous case, only large-width bumps are stable.

**Fig. 16:**
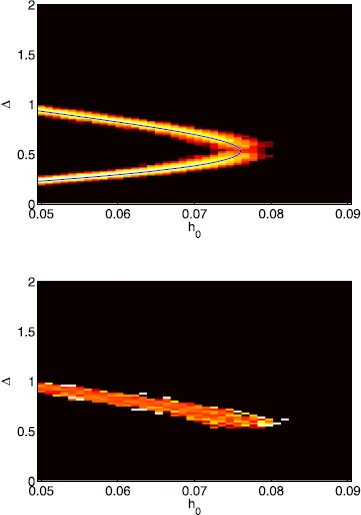
Probability distributions of bump widths for a Gaussian threshold distribution. *Top*: probability density of Δ values for a Gaussian threshold distribution. (*White* is high, *black* low.) The deterministic solution is superimposed in *blue*. *Bottom*: fraction of solutions which are stable. ($\mathit {Black}=0$, $\mathit{white}=1$.) Parameter values are $\alpha=5$, $B=0.76$, $\beta=3$, $\epsilon=0.002$, $\kappa=0.5$ and $\sigma^{2}=4$

When $\epsilon=0$ bumps only exist below a critical value of $h_{0}$, and a branch of stable bumps coalesces with a branch of unstable bumps at this critical value when $h_{0}$ is varied. Figure 16 shows results for a Gaussian threshold when we change $h_{0}$ for $\epsilon =0.002$. We again observe two solution branches that only exist below a critical value of $h_{0}$. Each solution branch is smeared out compared to the homogeneous limit, indicating a probability distribution that has a finite and non-zero width.

**Fig. 17:**
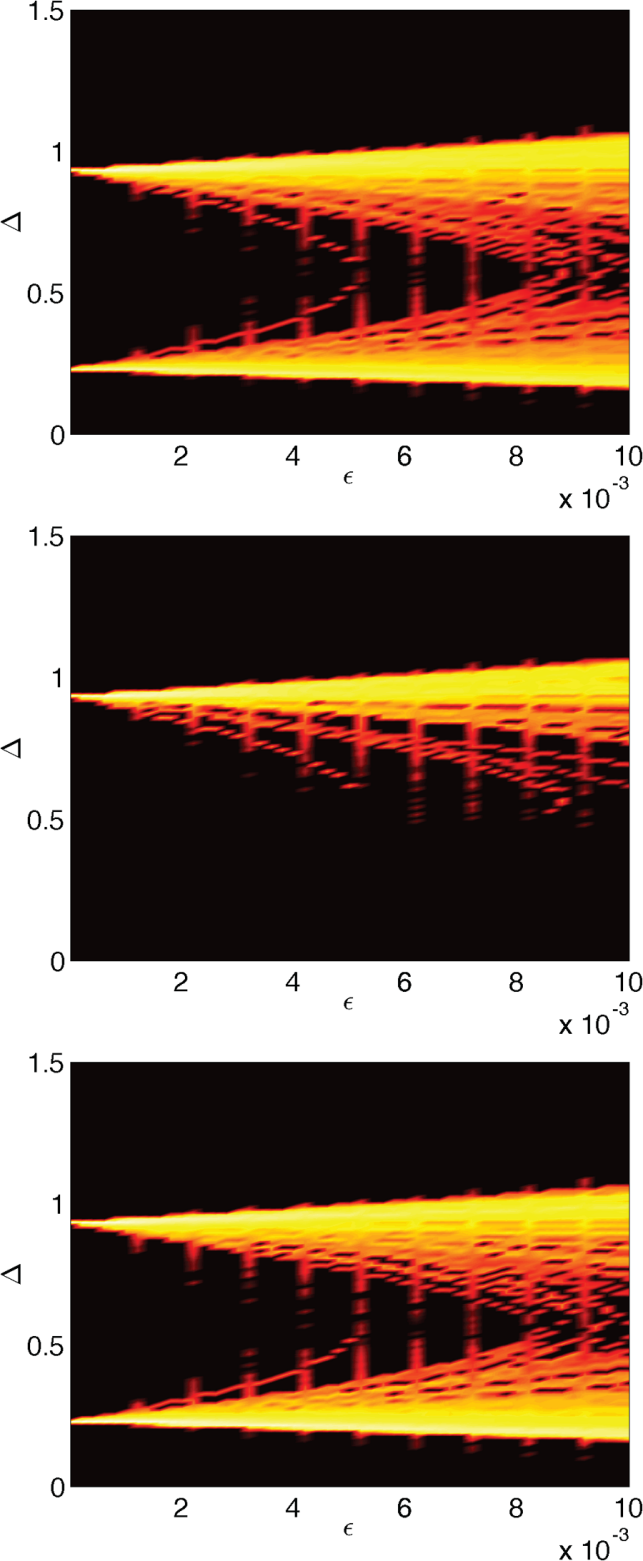
Probability distributions of bump widths for a non-Gaussian threshold distribution. Logarithm of the probability of the bump width Δ when the local probability distribution is given by a shifted exponential and the covariance function is Gaussian: all bumps (*top*), stable bumps (*middle*), unstable bumps (*bottom*). Parameter values are $\sigma=1$, $\mu =-1$, $\lambda=1$, $N=32$, $\kappa=1$, $L=2 \pi$ and $h_{0}=0.05$

Using the shifted exponential distribution for the threshold we obtain the results plotted in Fig. 17. We again observe two solution branches that emerge from the solutions in the homogeneous case as *ϵ* increases, with the stable solutions confined to the upper branch. In contrast to the Gaussian case in Fig. 15 the two solution branches do not grow symmetrically around the values of the homogeneous case. This is a manifestation of the highly skewed character of the exponential distribution compared to the symmetric Gaussian distribution. The probability distribution of the widths also exhibits much more structure compared to the Gaussian case.

**Fig. 18:**
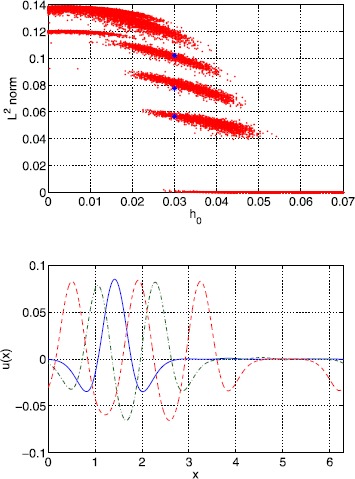
Multibumps. *Top*: $L^{2}$ norm of stable steady states of (3) for a Gaussian threshold distribution, with many different initial conditions, different realisations of $h(x)$, and various $h_{0}$. Solutions indicated by *blue stars* are shown in *the bottom panel*. *Bottom*: typical stable steady states solutions of (3) with 1, 2 and 3 bumps, for $h_{0}=0.03$. Their $L^{2}$ norms increase as the number of bumps increases (*blue dots* in *the top panel*). These three solutions correspond to three different realisations of $h(x)$. Parameter values are $\alpha=15$, $B=0.76$, $\beta=9$, $\epsilon =0.003$, $\kappa=0.5$ and $\sigma^{2}=4$

We know that for a Heaviside firing rate, Mexican-hat connectivity, and a constant firing threshold, multibump solutions cannot exist [39]. However, breaking the translational invariance by using a heterogeneous threshold does allow such solutions to exist. We show an example of this behaviour in Fig. 18, where the $L^{2}$ norms of stable steady states of (3) are shown as a function of $h_{0}$. Each point corresponds to a different realisation of $h(x)$ and a different initial condition. We clearly see different “bands”, each identified with an integer number of bumps in a solution, and the $L^{2}$ norm of these solutions increases as the number of bumps increases, in the same way as seen for systems with a smooth firing-rate function and homogeneous threshold [40–42], or with an oscillatory coupling function [39].

## Conclusion

In this paper we have explored the role of threshold noise on travelling fronts and bumps in a simple neural field model with a Heaviside nonlinearity. For a frozen form of disorder and a given realisation of a spatial threshold the standard rules of calculus apply and we have exploited the interface approach of Amari to obtain exact results about solution properties for both existence and stability. The theory that we have developed is not restricted to any special choice of distribution for describing the threshold noise, apart from that sample trajectories be differentiable in the mean square sense. It is worth noting that the stochastic threshold model presented here is formally equivalent to already published stochastic nonlinear integro-differential equations with constant threshold [6–14] when we employ the transformation $v(x,t)=u(x,t)-\epsilon g(x)$. However, our approach permits the analysis of strong noise (see e.g. [20]) and hence will allow us to move beyond perturbative expansions.

Theoretical predictions have been shown to be in excellent agreement with numerical simulations of both Gaussian and non-Gaussian threshold models. As such we have a viable mathematically tractable model of a noisy neural tissue that would be of interest to explore in a variety of more neurobiologically relevant scenarios. For example, temporal correlations in excitability of neural tissue would be expected to strongly affect wave propagation and could be easily modelled with an appropriate choice for $h=h(x,t)$. To investigate the consequences for wave speed all that would be required would be a minimal extension of the formula for interface dynamics (13) with the replacement of the numerator $u_{t}$ according to $u_{t} \rightarrow u_{t} - h_{t}$. Moreover, the inclusion of a linear adaptation current, as is common for describing spike frequency adaptation, would allow the study of travelling pulses as well as fronts, building on the deterministic approach in [43] and more recently in [44] for understanding cortical waves in epilepsy. The work here can also be extended to planar systems, allowing the study of spiral waves [45] and an investigation of how noise levels could be used to control the scale and size of a spiral, as suggested in [46]. These are topics of current investigation and will be reported upon elsewhere.

## Appendix A: Non-Gaussian Quenched Disorder

In the following, we will describe the iterative approach that we have used to generate non-Gaussian quenched disorder. The algorithm is based on [30]. We will use the more general form of the Karhunen–Loève decomposition in (7). To translate our findings for the $\alpha_{m}$ to the $\beta_{m}$ and $\gamma_{m}$ in (9) we note that this can be achieved by relabelling, i.e.

35 $$ \{\alpha_{1}, \alpha_{2}, \alpha_{3}, \alpha_{4},\ldots\}=\{\beta _{1},\gamma _{1}, \beta_{2},\gamma_{2},\ldots\} . $$

In the following it is convenient to introduce the notation $\alpha _{m}^{(k,l)}$, which denotes the *m*th expansion coefficient at the *k*th iteration for the *l*th realisation of the quenched disorder. To initialise the scheme, we generate *M* sets of uncorrelated $\alpha _{m}^{(0,l)}$, $l=1,\ldots,M$, drawn from the prescribed non-Gaussian distribution $\phi(g)$ shifted to mean zero and scaled to unit variance. A convenient way for doing this is to use inverse transform sampling based on the cumulative distribution function $F_{\phi}$ of $\phi (g)$. Strictly speaking the $\alpha_{m}^{(0,l)}$ could be generated from any mean zero and unit variance distribution, but starting with the prescribed distribution $\phi(g)$ might speed up convergence. For numerical purposes, the sum in (7) will only contain *N* terms. One method to determine *N* is to impose the condition

36 $$ \max_{y} \Biggl\{ \int_{-\infty}^{\infty}\Biggl\vert C(x,y)-\sum _{m=1}^{N} \lambda _{m} e_{m}(x) e_{m}(y) \Biggr\vert \,\mathrm {d}x \Biggr\} < \varepsilon, $$

i.e. that the maximal $L_{1}$ norm of the difference between the given covariance function and its approximation is smaller than some $\varepsilon>0$. Once *N* is fixed, each iteration step proceeds as follows:

1. Generate *M* samples of the quenched disorder
  37 $$ g^{(k,l)}(x)=\sum_{m=1}^{N} \sqrt{ \lambda_{m}} \alpha_{m}^{(k,l)} e_{m}(x) ,\quad l=1,\ldots, M , $$
  where $g^{(k,l)}$ denotes the *l*th realisation of the quenched disorder at the *k*th iteration. Note that because the $\alpha _{m}^{(k,l)}$ are uncorrelated with unit variance, the numerical error in determining the covariance function only depends on *N* (to satisfy (8)) and on *M* (for high-fidelity averaging).
2. Numerically determine the cumulative distribution function
  38 $$ \widetilde{F}^{(k)}_{g}(y)=\frac{1}{M} \sum _{l=1}^{M} \mathbb{I} \bigl( g^{(k,l)}(x) \leq y \bigr) , $$
  where $\mathbb{I}$ represents the indicator function, i.e. $\mathbb {I}(A)=1$ if *A* is true, otherwise $\mathbb{I}(A)=0$. The cumulative distribution function $\widetilde{F}^{(k)}_{g}(y)$ generally does not agree with the prescribed function $F_{\phi}$. The next two steps alleviate this problem.
3. Map the simulated values $g^{(k,l)}(x)$ to follow $F_{\phi}$:
  39 $$ h^{(k,l)}(x)=F_{\phi}^{-1} \circ \widetilde{F}^{(k)}_{g} \bigl(g^{(k,l)}(x) \bigr) . $$
4. Compute new values $\alpha_{m}^{(k+1,l)}$ as
  40 $$ \alpha_{m}^{(k+1,l)}=\frac{1}{\sqrt{\lambda_{m}} } \int_{0}^{L} \bigl( h^{(k,l)}(x)-\mathbb{E} \bigl(h^{(k,l)}(x)\bigr) \bigr) e_{m}(x) \,\mathrm {d}x . $$
  The mean of $\alpha_{m}^{(k+1,l)}$ is zero by construction, but the variance is unequal from one due to (39). Is therefore necessary to scale the $\alpha_{m}^{(k+1,l)}$ to have unit variance.
5. While the $\alpha_{m}^{(k+1,l)}$ now give rise to the prescribed probability distribution $\phi(g)$, they are correlated due to the mapping in (39). To decorrelate them, we use an iterative product-moment orthogonalisation technique.
  - Arrange the values of $\alpha_{m}^{(k+1,l)}$ into a $(M\times N)$ matrix *A*, i.e. the *m*th column contains the realisations of $\alpha _{m}$. The goal is to re-arrange each column such that correlations between columns are minimised. This is equivalent to having minimal correlations between draws of the $\alpha_{m}$.
  - Compute the $N \times N$ covariance matrix $C(A)$ of *A*.
  - Because $C(A)$ is positive definite, we can employ a Cholesky decomposition, i.e. $C(A)=G^{T}G$. Therefore, the new matrix $H=AG^{-1}$ is uncorrelated.
  - Reorder the entries in *A* to follow the rankings in *H*.
  Repeating the steps (a)–(d) will decrease the correlations of the $\alpha_{m}^{(k+1,l)}$ as required.

## Appendix B: Local Probability Distributions

We here provide details of the three zero-mean local probability distributions used in this study. We consider a Gaussian distribution

41 $$ \phi(x)=\frac{1}{\sqrt{2 \pi\sigma^{2}}} \exp \biggl[-\frac{x^{2}}{2 \sigma ^{2}} \biggr] ,\quad -\infty< x < \infty,\sigma>0 , $$

a highly skewed shifted exponential distribution

42 $$ \phi(x)=\exp \bigl[-(\lambda x+1) \bigr] ,\quad -\frac{1}{\lambda} \leq x < \infty, \lambda\in\mathbb{R} , $$

and a piecewise linear distribution with compact support

43 $$ \phi(x)= \textstyle\begin{cases} \alpha(L+x) , &-L \leq x \leq-B , \\ \alpha(L-B) , &-B \leq x \leq B , \\ \alpha(L-x) , &B \leq x \leq L , \end{cases}\displaystyle \quad 0 < B < L, \alpha>0 . $$

We refer to the last distribution as bump distribution. By demanding that it is normalised, we find $\alpha=1/(L^{2}-B^{2})$. The variances of the exponential and bump distribution are given, respectively, by

44 $$ \sigma^{2}_{\mathrm{SE}}=\frac{1}{\lambda^{2}} ,\qquad \sigma^{2}_{\mathrm{bu}}= \frac{L^{2}+B^{2}}{6} . $$
